# Founding Editorial — Child Health and Human Development

**DOI:** 10.1100/tsw.2003.21

**Published:** 2003-04-14

**Authors:** Joav Merrick

**Affiliations:** National Institute of Child Health and Human Development, Office of the Medical Director, Division for Mental Retardation, Ministry of Social Affairs, Box 1260, IL-91012 Jerusalem, and Zusman Child Development Center and Division of Community Health, Ben Gurion University, Beer-Sheva, Israel

## Abstract

The period of life called childhood is of worldwide interest, and is nicely illustrated by numerous stories about childrens life around the globe in a recent series of books published by John Wiley & Sons as part of the Open University course on “Childhood”[1,2]. Adolescence and later adulthood are also important parts of the human development that shape us and our future generations. In addition to genetics, the conditions and environment during our first few years of life will have a binding impact on the development taking place years ahead concerning our achievements in life, our accomplishments, and our health.

### CHILDHOOD OVER TIME

The French historian Philippe Aries[3] made us aware about the sentimentalization of childhood emerging in the $19^{th}$ and $20^{th}$ centuries. Before this time, children were just perceived as "small adults", which he documented with his analysis of art over time, where children indeed are depicted as small adults.

So slowly in the last 2 centries, childhood and later adolescence were introduced as separate entities and periods with their own development, concerns, and problems. We have therefore seen pediatrics, adolescent medicine, and even geriatrics emerge as specialties concerned with different aspects of human development.

This last century also saw advances in public health with decreased infant and maternal mortility in the Western World (or sometimes called the North), but while we enter this new century, the Developing World (or South) is still struggeling with disease and health concerns due to lack of resources. Since the global expansion of AIDS (Aquired Immunodeficiency Syndrome), mortality has increased (so far an estimated 20 million people have died due to AIDS related causes) and resulted in a growing number of children becoming orphans[4]. It is estimated that by year 2010, AIDS will leave 20 million children alone in Africa without one or both parents, which is double the current situation of 11 million orphaned children[4]. At present, 33.5

*Principal Editor, Child Health and Human Development
©2003 with author.

million people globally are HIV infected, which is estimated to increase to 45 million by year 2010[4] and most will be unable to afford the treatment.

Poverty and human development were the themes of a research project undertaken by the World Bank and published in three books[5,6,7]. This study will tell you the case studies from 14 countries. In all three books, information was gathered from more than 60,000 poor men and women from 60 countries; the true experts on poverty with many stories to tell together with relevant and important observations. The study is different from other poverty studies in the fact that participatory and qualitative research methods were used. The voices of the poor from the whole world can be heard from each page you read. So with progress and positive development, we still have to struggle with poverty and the results of poverty. Poverty is today even in developed countries like U.S. and Israel (about 25% of children living in poverty) a major public health problem of a magnitude that is markedly different than Scandinavian countries (3%)[8].

### CHILD HEALTH AND HUMAN DEVELOPMENT

The *Child Health and Human Development (CHHD)* domain is now an online journal within TheScientificWorldJOURNAL, publishing peer-reviewed, original research papers, reviews, short communications, case reports, methods papers, commentaries, letters to the editor, and book reviews at no cost to contributing authors of the international scientific community. All papers in final form are published immediately upon acceptance after peer review, and indexed within leading bibliographic services, including MedLine.

*Child Health and Human Development* is a multidisciplinary journal that will publish original research papers in the broad area of child health and human development. The journal provides an international forum for child public health issues, health and social policy, service aspects, developmental aspects, epidemiology, family and social issues, psycho-social pediatrics, community pediatrics, developmental pediatrics, behavioral pediatrics and ambulatory pediatrics, and human development over the whole age spectrum.

We would like for this journal to pursue a broad research agenda on a global basis, addressing questions ranging from policy to practice, and spanning the developmental spectrum from human genetics, infancy and early childhood, to adolescence and adulthood. We hope to generate new knowledge, to link research to practice and policy, to help build networks of research collaborations and move science forward in our field.

## EDITORIAL BOARD
# Child Health and Human Development — A Domain of TheScientificWorldJOURNAL

### PRINCIPAL EDITOR

**Joav Merrick, M.D., D.M.Sc.**
Professor
National Institute of Child Health and Human Development and Office of the Medical Director, Ministry of Social Affairs, Jerusalem, Israel

### ASSOCIATE EDITOR

**Hatim A. Omar, M.D.**
Associate Professor
Adolescent Medicine and Young Parent Programs, Department of Pediatrics and Adolescent Medicine, University of Kentucky Medical Center, Lexington

### EDITORIAL BOARD

**David L. Bennett, A.O., F.R.A.C.P.**
Professor
Department of Adolescent Medicine, Children's Hospital Westmead, Sydney, Australia

**Bengt Lindström, M.D., Ph.D., Dr.P.H.**
Professor
Nordic School of Public Health, Gottenburg, Sweden

**Mohammed Morad, M.D.**
Lecturer
Clalit Health Services and Faculty of Health Sciences, Ben-Gurion University of the Negev, Beer-Sheva, Israel

**Colin Pritchard, M.D.**
Professor
Institute of Health and Community Studies, Bournemouth University, Bournemouth, U.K.

**Daniel T.L. Shek, Ph.D., B.B.S., J.P.**
Professor
Department of Social Work, The Chinese Universityof Hong Kong, Shatin

**Yoshiyuki Suzuki, M.D.**
Professor
Clinical Research Center, Nasu Institute for Developmental Disabilities, International University of Health and Welfare, Otawara, Japan

**Daniel A. Vardy, M.D., M.Sc.**
Professor
Clalit Health Services and Faculty of Health Sciences, Ben-Gurion University of the Negev, Beer-Sheva, Israel

### REVIEW BOARD

**Eli Carmeli, P.T., Ph.D.**
Associate Professor
Department of Physical Therapy, Sackler School of Medicine, Tel Aviv University, Israel

**Daniel Chemtob, M.D., M.P.H., D.E.A.**
Director
Department of Tuberculosis and AIDS, Public Health Services, Ministry of Health, Jerusalem, Israel

**Aharon Galil, M.D.**
Professor
Zusman Child Development Center, Soroka University Medical Center, Faculty of Health Sciences, Ben-Gurion University of the Negev, Beer-Sheva, Israel

**Mike Kerr, M.D.**
Senior Lecturer
Welsh Centre for Learning Disabilities, Cardiff, U.K.

**Shlomo Kessel, M.S.W.**
Director
Neve Michael Children's Village, Pardes Hanna, Israel

**Denis Weintraub, A.M., M.A.**
Faculty of Health Sciences, Ben-Gurion University of the Negev, Beer-Sheva, Israel
